# Interplay Between NLRP3 Inflammasome and Autophagy

**DOI:** 10.3389/fimmu.2020.591803

**Published:** 2020-10-09

**Authors:** Monika Biasizzo, Nataša Kopitar-Jerala

**Affiliations:** ^1^Department of Biochemistry, Molecular and Structural Biology, JoŽef Stefan Institute, Ljubljana, Slovenia; ^2^Jožef Stefan International Postgraduate School, Ljubljana, Slovenia

**Keywords:** NLRP3 inflammasome, autophagy, mitophagy, inflammation, inflammatory diseases

## Abstract

The NLRP3 inflammasome is cytosolic multi-protein complex that induces inflammation and pyroptotic cell death in response to both pathogen (PAMPs) and endogenous activators (DAMPs). Recognition of PAMPs or DAMPs leads to formation of the inflammasome complex, which results in activation of caspase-1, followed by cleavage and release of pro-inflammatory cytokines. Excessive activation of NLRP3 inflammasome can contribute to development of inflammatory diseases and cancer. Autophagy is vital intracellular process for recycling and removal of damaged proteins and organelles, as well as destruction of intracellular pathogens. Cytosolic components are sequestered in a double-membrane vesicle—autophagosome, which then fuses with lysosome resulting in degradation of the cargo. The autophagy dysfunction can lead to diseases with hyperinflammation and excessive activation of NLRP3 inflammasome and thus acts as a major regulator of inflammasomes. Autophagic removal of NLRP3 inflammasome activators, such as intracellular DAMPs, NLRP3 inflammasome components, and cytokines can reduce inflammasome activation and inflammatory response. Likewise, inflammasome signaling pathways can regulate autophagic process necessary for balance between required host defense inflammatory response and prevention of excessive and detrimental inflammation. Autophagy has a protective role in some inflammatory diseases associated with NLRP3 inflammasome, including gouty arthritis, familial Mediterranean fever (FMF), and sepsis. Understanding the interregulation between these two essential biological processes is necessary to comprehend the biological mechanisms and designing possible treatments for multiple inflammatory diseases.

## Introduction

Inflammatory innate immune responses are essential in host defense against pathogens. Similar responses and pathways protect the host from the microbial infections and endogenous danger signals. However, dysregulated and excessive inflammatory reaction can inflict tissue damage and inflammation is regarded as an underlying cause of some human diseases and disorders (1–3).

One of the main inflammatory pathways leading to the development of inflammatory diseases involves activation of inflammasome, a multi-protein complex that intensify inflammatory responses to both pathogen and endogenous activators (4–6). The NLRP3 (NOD-, LRR-, and pyrin-domain containing protein 3) inflammasome is activated by a variety of stimuli and is the most extensively studied inflammasome complex (7). In the last decade, the research has been focused on uncovering the mechanism of NLRP3 inflammasome activation and its regulation (3, 8, 9). An ever-growing number of studies have demonstrated interregulation of inflammasomes and autophagy.

Autophagy is an intracellular process important for recycling of damaged proteins and organelles, as well as destruction of intracellular pathogens (10, 11). The autophagy dysfunction can lead to inflammatory diseases (e.g., Inflammatory bowel disease) with hyperinflammation and excessive activation of inflammasomes (12–14). Similarly, innate immune responses initiate autophagy in response to infectious threats and inflammatory signals can upregulate autophagy to suppress excessive response and protect the host (14, 15).

It is important to understand this crosstalk between inflammation and autophagy, as it applies to various inflammatory diseases and this review will focus on the current available information about this mutual regulation that exist between NLRP3 inflammasome and autophagy.

## The NLRP3 Inflammasome

The inflammasome was first described as large multimeric protein complex required for caspase-1 processing and activation of inflammatory cytokines interleukin-1β (IL-1β) by Martinon et al. (16). The NLRP3 inflammasome consists of a sensor protein (NLRP3), an adaptor protein ASC (apoptosis-associated Speck-like protein containing CARD) and an effector caspase-1. NLRP3 is comprised of three domains: amino-terminal pyrin domain (PYD), central NACHT domaim (domain present in NAIP, CIITA, HET-E, and TP-2) and carboxy-terminal leucine-rich repeats (LRR). The central NACHT domain has ATPase activity that is vital for NLRP3 self-association and oligomerization (17). The LRR domain in NOD family members is thought to form inhibitory interactions with NACHT domain, that are relieved by recognition of stimulating ligands *via* LRR domain (18). However, a recent study has suggested that the LRR domain in NLRP3 does not have autoinhibitory function, given that variants lacking the LRR domain were not constitutively active (19). The LRR domain in NLRP3 was demonstrated to be dispensable for canonical NLRP3 activation (19), however different studies have suggested that LRR domain is responsible for interactions of NLRP3 with other proteins and hence necessary for inflammasome and ASC-speck formation (20, 21). The PYD domain allows NLRP3 to interact with other inflammasome proteins and these interactions are regulated by its phosphorylation (22).

ASC has two protein interaction domains, an amino-terminal PYD and carboxy-terminal CARD. Upon detecting specific stimuli sensor protein NLRP3 interacts with ASC *via* homotypic PYD-PYD domain interaction (23) and nucleates ASC into prion-like filaments, thereby forming a single ASC “speck” within activated cell. It was demonstrated that trimerization but not dimerization of NLRP3 pyrin domain induces caspase-1-dependent IL-1β maturation and release. Since foldon-induced trimerization did not lead to ASC speck formation it cannot be excluded that wild-type NLRP3 forms higher oligomer species (24). Adaptor protein ASC recruits pro-caspase-1 through CARD-CARD domain interactions (25, 26). Pro-caspase-1 undergoes proximity-induced autoproteolytic cleavage at the linker between large (p20) and small (p10) catalytic subunits to generate transient species p33/p10 (p33 consists of the CARD and p20), which remains bound to ASC and is proteolytically active (27). Further processing between the CARD and p20 releases heterotetramer p20/p10 from the inflammasome. The released p20/p10 heterotetramer is unstable in cells and its proteolytic activity is terminated (27). Active caspase-1 further cleaves proinflammatory cytokines of IL-1 family, such as IL-1β and IL-18 (28, 29).

Recently, serine/threonine NIMA-related kinase 7 (NEK7) was shown to be essential for NLRP3 inflammasome activation and appears to be a core component specific to the NLRP3 inflammasome (20, 21, 30). NEK7 specifically interacts with NLRP3 *via* LRR and NACHT domain independently of its kinase activity, but not with other inflammasome sensors NLRC4 (NOD-, LRR-, and CARD-containing 4) or AIM2 (absent in melanoma 2) (20, 21). The NLRP3 inflammasome activation triggers the interaction of NLRP3 with NEK7, leading to the inflammasome assembly, the ASC speck formation and caspase 1 activation. The NEK7-NLRP3 interaction was shown to be dependent on potassium efflux (20).

### Activation of NLRP3 Inflammasome

The NLRP3 inflammasome activation is considered to be a two-step process that requires two signals: (i) the first priming signal and (ii) the second NLRP3 activation signal. The priming step triggers nuclear factor-κB (NF-κB)-dependent upregulation of NLRP3 and pro-IL-1β expression (31) and lowers the activation threshold of NLRP3 by additional post-translational modification (PTMs) (8, 32). The second step is recognition of NLRP3 activator, which induces NLRP3 activation and inflammasome formation. Most pattern-recognition receptors (PRR) have limited specificity for one or few related pathogen-associated molecular patterns (PAMPs) or damage-associated molecular patterns (DAMPs). However, The NLRP3 is activated by wide range of bacterial, viral, and fungal PAMPs and endogenous DAMPs, such as pore-forming toxins, crystals, aggregates (such as β-amyloid), extracellular ATP, and hyaluronan (32). It is generally agreed that detection of such a diversity of agents cannot be direct (23, 33, 34).

**Figure 1:**
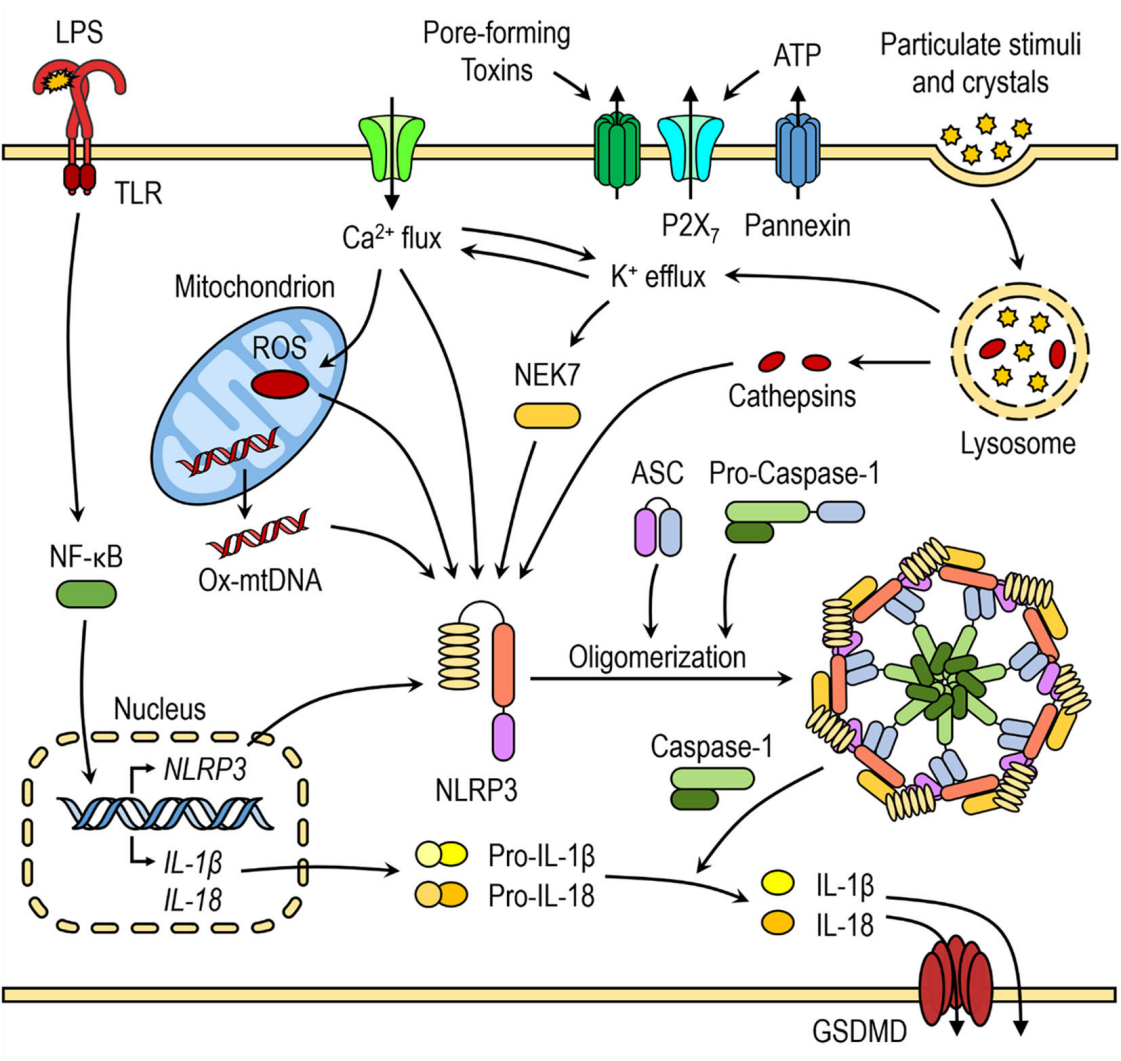
Canonical NLRP3 inflammasome activation. The priming step triggers nuclear factor-κB (NF-κB)-dependent upregulation of NLRP3 and pro-inflammatory cytokine expression. The second step is recognition of NLRP3 activator, which induces NLRP3 activation and inflammasome formation. Several molecular mechanisms have been proposed for NLRP3 activation, including K^+^ efflux, Ca^2+^ flux, lysosomal destabilization, mitochondrial dysfunction and release of mtROS and mtDNA.

Recently, several in-depth reviews discussed molecular mechanisms of NLRP3 inflammasome activation (8, 9). Several molecular mechanisms, most of which are not mutually exclusive, have been proposed for NLRP3 inflammasome activation, including efflux of potassium ions (K^+^), flux of calcium ions (Ca^2+^), lysosomal disruption, mitochondrial dysfunction, metabolic changes, and trans-Golgi disassembly (Figure 1) (8). Many of NLRP3 activators, such as nigericin, ATP, pore-forming toxins, and particulate stimuli, are known to induce potassium efflux and decrease intracellular potassium levels, which is required for direct binding of NEK7 to NLRP3 inflammasome (20, 35). Furthermore, low extracellular concentrations of K^+^ were shown to be sufficient for NLRP3 inflammasome activation in the absence of an NLRP3 agonist, whereas high extracellular concentration of K^+^ prevented its activation (36, 37). Ca^2+^ signaling and mobilization were demonstrated to have a critical role in the NLRP3 inflammasome activation, however it is often coordinated with K^+^ efflux (36, 38). Blocking Ca^2+^ mobilization inhibited the NLRP3 inflammasome assembly and activation in macrophages (38, 39).

Mitochondrial dysfunction and release of mitochondrial ROS (mtROS) and mitochondrial DNA (mtDNA) are another important triggers for NLRP3 inflammasome activation and some of NLRP3 activators induce increased mtROS and cytosolic ROS generation. Even though the exact mechanism of NLRP3 inflammasome activation mediated by generation of mitochondrial ROS is not yet fully elucidated, several hypotheses have been suggested. The first hypothesis involves interaction between thioredoxin-interacting protein (TXNIP) and NLRP3 after an increase in ROS caused by NLRP3 activators, such as MSU. TXNIP was shown to be crucial for redox-stress mediated NLRP3 inflammasome activation (40). Another hypothesis suggests that NLRP3, which is mostly localized in ER in resting conditions, translocates to mitochondria and mitochondria-associated ER membranes (MAMs) upon NLRP3 inflammasome activation by its agonists (nigericin, MSU, or alum), placing it in close proximity to newly formed mitochondrial ROS (41).

Activators, such as crystalline structures and aggregates cause lysosomal destabilization and rupture (42, 43). Cathepsin B, released from ruptured lysosomes was shown to directly bind to NLRP3 inflammasome and contribute to NLRP3 inflammasome activation (44). However, it was demonstrated that the lysosomal damage caused by Leu-Leu-OMe and NLRP3 particulate stimuli activates K^+^ efflux and Ca^2+^ influx, indicating that many NLRP3 activation pathways converge on either K^+^ and/or Ca^2+^ flux (36, 38, 45).

In persistent infections, such as tuberculosis caused by *Mycobacterium tuberculosis*, the NLRP3 inflammasome activation and the consequent IL-1β-initiated inflammatory response is regulated to maintain tissue integrity (46). Ineffective control of the infection leads to activation of adaptive immunity and the release of interferon-γ (IFN-γ) by lymphocytes. IFN-y stimulated macrophages are triggered to express antimicrobial effectors, including inducible nitric oxide synthase (iNOS). The resulting nitric oxide (NO) inhibits bacterial growth, as well as suppresses the continual production of IL-1β by the NLRP3 inflammasome (46). The suppressive effect of IFN-γ and NO was specific to the NLRP3 inflammasome. NO was suggested to post-translationally modify the NLRP3 protein by thiol-nitrosylation, which inhibits ASC oligomerization, the NLRP3 inflammasome assembly and processing of IL-1β (46, 47). NO-mediated inflammasome inhibition therefore represents an important mechanism to prevent tissue damage during persistent and chronic infection (46).

In response to cytosolic LPS, a key component of Gram negative bacteria, caspase-1 can be activated independently of canonical inflammasomes (48). Caspase-11, an inflammatory caspase closely related to caspase-1, directly binds cytosolic LPS. This interaction triggers self-oligomerization and activation which leads to caspase-1 activation, as well as pyroptotic cell death (49). Pyroptosis is an inflammatory form of a programmed cell death following activation of caspase-1 and caspase-11. Active caspase-1 and caspase-11 may eventually cleave gasdermin D (GSDMD) which subsequently leads to GSDMD self-oligomerization at cell membrane and pore formation (50–53). GSDMD membrane pores induce ionic flux and NLRP3 inflammasome activation (54).

### Bacterial Infection and NLRP3 Inflammasome Activation

Several infectious microbes were demonstrated to activate NLRP3 inflammasome. Earlier studies demonstrated that Gram-positive *Staphylococcus aureus* and *Listeria monocytogenes*, Gram-negative *Shigella sonnei*, and *Shigella flexneri*, but not Gram-negative *Salmonella typhimurium* and *Francisella tularensis* trigger NLRP3-dependent IL-1β secretion (55). *S. sonnei* induced IL-1β production through P2X7 receptor-mediated potassium efflux, reactive oxygen species generation, mitochondrial damage, and lysosomal acidification in LPS-primed primary murine macrophages (56).

Live intracellular *Mycobacterium tuberculosis* infection induced NLRP3-dependent IL-1β secretion and pyroptosis in human monocytes and macrophages (57, 58). *M. tuberculosis* was shown to damage host cell plasma membrane either during phagocytosis or following phagocytosis and phagosome rupture from cytosolic side. The damage caused by *M. tuberculosis* made plasma membrane permeable to both K^+^ and Ca^2+^ ions, which results in the NLRP3 inflammasome activation (58). *M. tuberculosis* was also shown to induce NLRP3-dependent pyroptosis in human monocytes which enables bacteria to spread immediately after cell death (58) However, a different study reported the NLRP3-dependent necrotic cell death that is independent of caspase-1 in primary human macrophages and THP-1 monocytes (59).

*Salmonella typhimurium* infection of human macrophages indirectly activated NLRP3 inflammasome (60). *S. typhimurium* infection leads to *Salmonella* sequestration within the *Salmonella*-containing vesicles, which are eventually ruptured and bacteria are released into the cytosol (60). In the cytosol bacterial LPS activated caspase-4/caspase-11 and induced pyroptosis and consequently activated NLRP3 inflammasome (60). However, NLRP3 had a limited role in *Salmonella* infection in mice, as a bacterial burden, a pro-inflammatory cytokine level, and an organ pathology did not differ between NLRP3 deficient and wild-type mice (61). Furthermore, NLRP3 inhibitor suppressed IL-1β release and pyroptosis in human macrophages only when NLRC4 function was also ablated (60).

## Autophagy

Autophagy, specifically macroautophagy, is an intracellular process important for cellular homeostasis and delivery of cytosolic constituents, including organelles, to lysosomes for degradation and amino acid recycling (62). Autophagy is regulated by a wide range of proteins that are the products of autophagy-related genes (Atg), including Atg8 (MAP1LC3; also known as LC3), which is commonly used as a marker for visualizing and quantitating autophagosomes (63). Autophagy begins with sequestration of organelle or portion of cytoplasm by phagophore, a membranous structure that elongates to engulf cytoplasmic cargo, which results in formation of an autophagosome with a double membrane. The initiation of autophagosome formation begins with activation of ULK kinase complex (ULK1 or ULK2, ATG13, FIP200, ATG101), which targets a class III PI3K complex (Beclin1, VPS15, VPS34, ATG14) (64, 65). In the expansion stage the ATG12-ATG5-ATG16 complex is recruited to the autophagosome membrane, which facilitates the lipidation of LC3 that is required for the expansion of autophagic membranes (64). The resulting autophagosome the fuses with lysosome resulting in degradation of the cargo (66). As an important homeostatic mechanism for degradation and recycling of cytosolic components, autophagy enables generation of amino acids during starvation and elimination of damaged and dysfunctional organelles. As such, autophagy is up-regulated in response to amino acid starvation and damaged organelles, including mitochondria (mitophagy) (65, 67).

Autophagy induction represents one of the most primitive examples of the innate immune responses and act as the first line of defense during intracellular pathogen infection, including *Mycobacterium tuberculosis* (68), *Shigella flexneri* (69), and *Salmonella typhimurium* (70). During infection autophagy is induced by Toll-like receptors that recognize PAMPs (71, 72). Autophagosomes engulf pathogens and direct them to lysosomes for degradation, which kill pathogens and enables to present pathogen components to the innate and adaptive immune system (73). Hence, autophagy up-regulation might enhance the clearance of some infectious pathogens. Several different pharmacological modulators were demonstrated to enhance or activate autophagy and restrict growth and/or decrease bacterial burden of *M. tuberculosis* in macrophages or in mice (74–78). Furthermore, autophagy regulates and is regulates by a wide range in cytokines (79).

## Autophagy Regulates NLRP3 Inflammasome Activation

**Figure 2:**
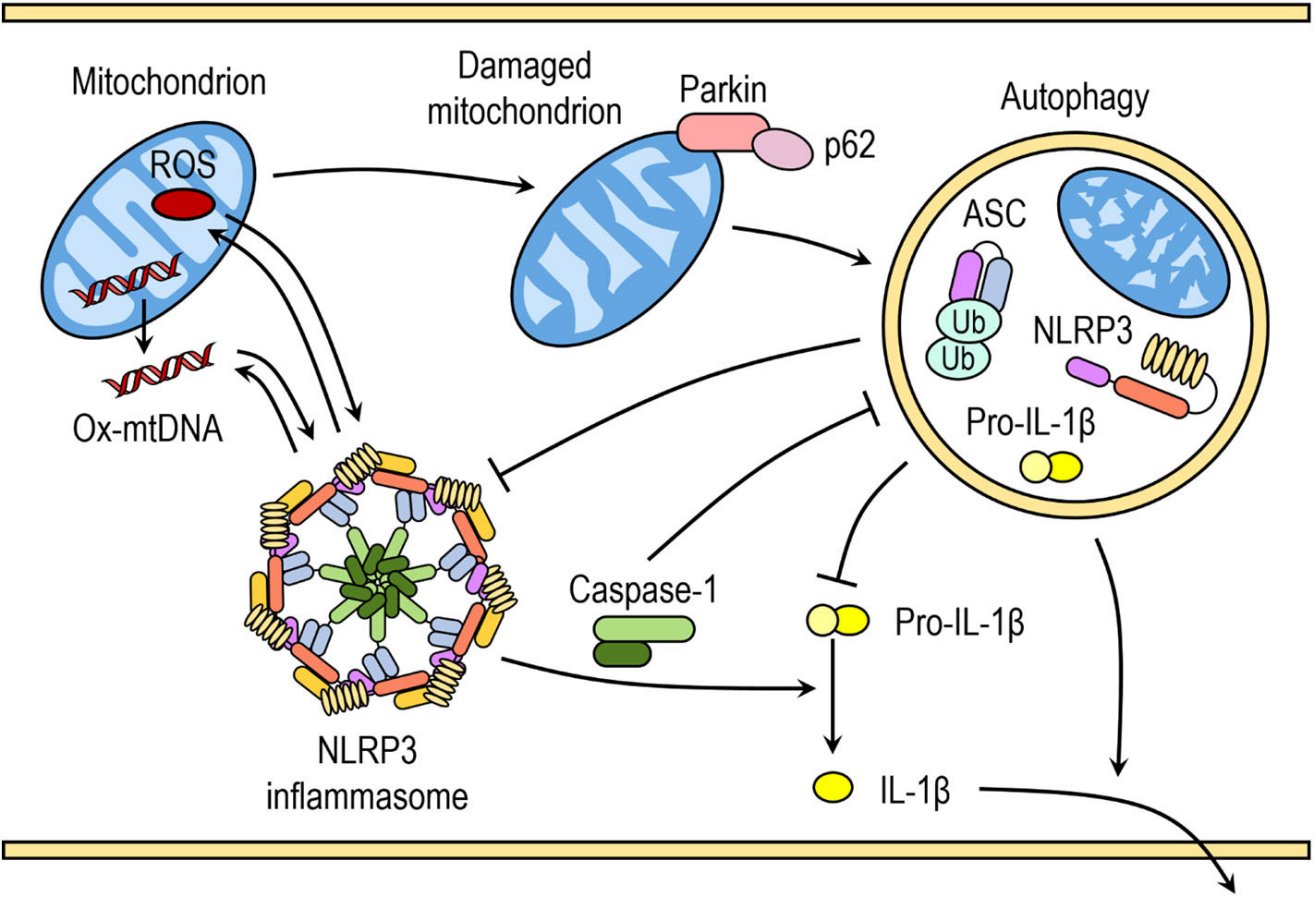
Crosstalk between inflammasomes and autophagy. Autophagy can negatively regulate NLRP3 inflammasome activation by removing endogenous inflammasome activators, such as ROS-producing damaged mitochondria, removing inflammasome components and cytokines. Autophagic machinery also has a role in unconventional secretion of IL-1β and thus regulates inflammatory response. Conversely, NLRP3 inflammasome activation regulates autophagosome formation through several different mechanisms. Crosstalk between inflammasomes and autophagy is necessary for balance between required host defense inflammatory response and prevention of excessive inflammation.

Saitoh et al. (80) were the first to demonstrate the link between autophagy, inflammasome activation and cytokines processing. The loss of autophagy-related protein ATG16L1 in mouse fetal liver-derived macrophages resulted in increased caspase-1 activation and IL-1β processing after endotoxin treatment. Similarly, IL-1β production in macrophages deficient in ATG7 protein or treated with autophagy inhibitor 3-methyladenine (3-MA) is enhanced in response to endotoxin (80). In the last decade numerous studies have further indicated that autophagy can regulate inflammasome activation, including the NLRP3 inflammasome activation, through various mechanisms (Figure 2) (81).

### Autophagic Removal of Endogenous NLRP3 Inflammasome Activators

Autophagy removes damaged organelles, such as mitochondria, leading to reduced release of mitochondrial-derived DAMPs and suppression of inflammasome activation. Zhou et al. demonstrated that increased ROS production in mitochondria by pharmacological inhibition of mitochondrial complex I and III is responsible for increased NLRP3-dependent caspase-1 activation and IL-1β release in monocytes and macrophages (41). Downregulation of voltage dependent anion channels (VDAC), which are required for ROS production, or ROS scavengers impaired and reversed NLRP3-mediated caspase-1 activation and IL-1β release in response to NLRP3 activators nigericin, MSU, alum, and silica (41). Similarly, inhibition with 3-methyladenine (3-MA) or loss of autophagy resulted in accumulation of ROS-producing mitochondria and subsequently enhanced inflammasome activation in response to NLRP3 activators (ATP, nigericin, and MSU), which was further reversed by ROS scavengers (41, 82, 83).

It was reported that mitochondrial ROS production and subsequent mitochondrial membrane permeability transition (MPT) leads to translocation of mtDNA into the cytosol after LPS and ATP treatment (82). Cytosolic mtDNA was suggested to directly or indirectly associate with NLRP3 and to contribute to downstream activation of caspase-1 in response to LPS and ATP (82, 84). Generation of mtROS can results in oxidized mtDNA, which enhanced NLRP3 inflammasome activation compared to normal mtDNA, suggesting that both mitochondrial ROS and mtDNA are important for NLRP3 inflammasome activation (84).

Disruption of autophagy by inhibitors or through downregulation of proteins with crucial role in autophagy can result in the accumulation of damaged mitochondria and increased concentration of mitochondrial ROS (41, 82). ROS-generating or damaged mitochondria are constantly removed by mitophagy, specialized form of autophagy, to maintain mitochondrial homeostasis. Damaged mitochondria with decreased membrane potential are marked with ubiquitination of outer membrane proteins by E3 ubiquitin ligase Parkin and tagged for autophagic disposal (85).

A recent study by Zhong et al. (86) suggested that SQSTM1/p62 was an essential mediator of mitophagic elimination of damaged mitochondria upon NLRP3 activation. The priming with LPS induces NF-κB-dependent p62 expression in macrophages and p62 ablation in macrophages enhances IL-1β production. Upon NLRP3 activation, p62 is recruited to damaged mitochondria, which requires Parkin-dependent decoration of damaged mitochondria with poly-ubiquitin chains. Damaged mitochondria due to NLRP3 activation are removed through p62-mediated mitophagy. Therefore, a Parkin-dependent clearance of p62-bound damaged mitochondria reduces NLRP3 inflammasome activation by NLRP3 activators in macrophages (86). This is supported by increasing number of studies demonstrating that impaired mitophagy enhances NLRP3 activation, whereas induction of mitophagy reduces NLRP3 activation (87–90). However, reduction of autophagy by itself does not seem to be sufficient to trigger inflammasome activation in the absence of NLRP3 activators (41, 82).

### Autophagy Targets NLRP3 Inflammasome Components

Another mechanism to prevent excessive inflammasome activation is through p62-dependent degradation of inflammasome components. Upon stimulation of NLRP3 inflammasome in monocytes, ASC is recognized by p62 and the NLRP3 inflammasome components, including NLRP3 and ASC, co-localize with autophagosomes, indicating that the NLRP3 inflammasome can be engulfed and degraded by autophagosomes (91). ASC in the assembled AIM2 inflammasome complex undergoes K63-linked polyubiquitination, which is recognized by p62 and similar mechanism could be involved in the NLRP3 inflammasome (91). The pharmacological inhibition of autophagy and loss of p62 greatly enhanced the NLRP3 inflammasome activation (91).

The tripartite motif (TRIM) family contains several RING finger domain-containing proteins and play an important role in innate immune response (92). Tripartite motif 20 (TRIM20, pyrin) was shown to interact with the NLRP3 inflammasome components, including NLRP3, ASC, caspase-1, and pro-IL-1β, thereby modulating their activity (93, 94). Pyrin was also demonstrated to organize autophagic machinery by serving as a platform for the assembly of ULK1, Beclin1, and ATG16L1 (95, 96). Therefore, pyrin was suggested to act as an autophagy receptor for delivery of the NLRP3 inflammasome components for autophagic degradation (95, 96). Correspondingly, a knockdown of pyrin spared NLRP3 degradation upon its activation in monocytes and conversely, overexpression of pyrin decreased levels of co-expressed NLRP3 in HEK293 cells, which was suppressed by inhibiting fusion between autophagosomes and lysosomes with Bafilomycin A1 (95). The NLRP3 degradation was dependent on ULK1 and Beclin1, establishing that disposal of NLRP3 was through autophagy (95).

Recently, it was suggested that NLRP3 phosphorylation mediates its inactivation in autophagy-dependent manner (97). NLRP3 activation is negatively regulated by tyrosine phosphorylation and protein tyrosine phosphatase non-receptor 22 (PTPN22) dephosphorylates NLRP3 upon its activation (98). It was reported that only phosphorylated NLRP3 interacted with p62 in ASC-dependent manner and was sequestered into phagophore. NLRP3 lacking phosphorylation site did not interact with p62 and was not sequestered into phagophore (97).

The NLRP3 inflammasome was shown to interact with autophagic machinery, co-localize with autophagosomes and inhibition of autophagic pathway caused enhanced NLRP3 inflammasome activation. This suggests that post-translational modifications of NLRP3 (such as phosphorylation and ubiquitination) and subsequent autophagic removal of inflammasome components upon their activation serves as a negative-feedback loop to prevent excessive inflammatory response (91, 96, 97, 99, 100).

Supporting this notion, a recent study by Han et al. (101) showed that autophagy induction with small molecules (kaempferol—Ka) promoted degradation of inflammasome components and reduced of inflammasome activation. Ka was shown to promote autophagy in microglia, as indicated by higher LC3-II production, which was abolished by 3-MA. The NLPR3 inflammasome assembly was disrupted due to reduced NLRP3 protein levels, which resulted in decreased caspase-1 activation and IL-1β production upon the NLRP3 inflammasome activation after Ka treatment (101). The NLRP3 downregulation was mostly due to autophagic degradation, which was suppressed by autophagy inhibitor 3-MA (101).

### Autophagy and IL-1β Signaling

Besides sequestering inflammasome components, autophagosomes have been shown to target IL-1β in macrophages following TLR activation (102). Pro-IL-1β is not expressed in resting macrophages and its induction requires stimulation, typically by a TLR ligand. Harris et al. (102) reported that induction of autophagy with rapamycin, a pharmacological inhibitor of mTOR, during or after LPS priming of macrophages leads to reduction of intracellular pro-IL-1β and subsequent release of mature IL-1β in response to NLRP3 activators. Consistently, autophagy inhibition increased IL-1β secretion as more pro-IL-1β was available in the cytosol (102). These data suggest a role for autophagy in intracellular degradation of pro-IL-1β, proposing another mechanism by which autophagy regulates inflammatory response. However, another study in dendritic cells has shown that IL-1β and IL-1α are polyubiquitinated and intracellularly degraded by proteasomes, not autophagosomes (103).

IL-1β lacks a classical signal peptide to direct its cellular exit *via* secretory vesicles and, thus, follows an unconventional pathway for secretion. Upon inflammasome activation in macrophages and dendritic cells, IL-1β and IL-18 may be released as a results of pyroptotic cell lysis mediated by GSDMD (50, 51, 104). However, neutrophils, dendritic cells, monocytes, and hyperactivated macrophages can in some cases release IL-1β without cell lysis, suggesting that other mechanisms for IL-1β release are necessary (104–106). Monteleone et al. (107) reported that IL-1β processing by caspase-1 is not only necessary but also sufficient for its secretion and only mature IL-1β is actively secreted by macrophages. Mature IL-1β was reported to co-localize with negatively charged phosphatidylinositol 4,5-bisphosphate (PIP2) in the plasma membrane (107). Relocation of mature IL-1β to PIP2-enriched plasma membrane microdomains was suggested to facilitate slow GSDMD-independent release in resting conditions and rapid GSDMD-dependent release upon inflammasome activation, regardless of immediate cell fate (107).

Besides GSDMD pathway, several studies have suggested that autophagy acts as unconventional secretion of IL-1β. Dupont et al. (108) showed that induction of autophagy by starvation or mTOR inhibitor pp242 strongly enhanced IL-1β secretion in response to conventional NLRP3 activators in macrophages. Likewise, inhibition of autophagy in human neutrophils after LPS stimulation reduced IL-1β secretion and increased intracellular IL-1β levels (109). Using a reconstituted model of IL-1β secretion downstream of inflammasome activation in non-macrophage cells it was demonstrated that mature IL-1β localizes in the lumen between two membranes of autophagosomes (110). Furthermore, it was suggested that IL-1β is translocated across a membrane into a vesicle precursor of phagophore at a very early stage in the development of the organelle and that entry of IL-1β into this vesicle carrier requires conformational flexibility (110).

At present, there are discrepancies in the results between different studies showing autophagy enhancing and reducing IL-1β release. Study from Zhang et al. (110) gives an interesting insight on how can autophagy be necessary for IL-1β release without its degradation. It was also observed that smaller amount of mature IL-1β may be engulfed by the autophagosomes and may be degraded during autophagosome maturation (110). The regulation of IL-1β release by autophagic machinery is obviously complex and requires further investigation, and may be dependent on specific conditions such as cell type, inflammasome activator, and autophagy inducer/inhibitor.

## NLRP3 Inflammasome Regulation of Autophagy

The NLRs have been reported to interact with autophagy proteins which provides a mechanism for direct NLR regulation of autophagy. It was shown that several inflammasome-forming NLRs, including NLRP3 can interact with Beclin 1, a protein involved in autophagy initiation, through NACHT domain (111). NLRP3 was shown to modulate autophagy. Silencing core molecules of the NLRP3 inflammasome complex in human macrophages reduced the autophagy response after infection with *Pseudomonas aeruginosa*, however it enhanced the macrophage-mediated killing of internalized *P. aeruginosa* (112). Consistently, overexpression of core molecules of NLRP3 inflammasome elevated autophagy and increased amount of the LC3-II protein in human macrophages infected with *P. aeruginosa* (112). Furthermore, silencing of the NLRP3 downregulated autophagy and LC3-I conversion to LC3-II induced by MSU in osteoblasts (113).

However, the NLRP3 inflammasome was also reported to negatively control autophagy in microglia cells after stimulation with neurotoxic prion peptide PrP106-126, which activates the NLRP3 inflammasome (114). Furthermore, a few other studies have shown that NLRP3-deficient mice have increased autophagy levels at baseline and under stress conditions, such as hypoxia or hyperoxia in different tissues and epithelial cells (115, 116). The discrepancies between different studies could be due to different NLRP3 inflammasome activators, which could also activate other inflammasome complexes and signaling pathways.

Besides NLRP3, caspase-1 was also reported to regulate the autophagic process through cleavage of other substrates (117, 118). Yu et al. (118) demonstrated that inflammasome activation leads to a rapid caspase-1-dependent block of mitophagy in macrophages, which results in accumulation of mtDNA and dysfunctional mitochondria. Upon inflammasome activation caspase-1-mediated cleavage of Parkin contributes to caspase-1-dependent block of mitophagy in bone marrow-derived macrophages and this leads to accumulation of mtDNA and dysfunctional mitochondria, which is attenuated in cells expressing cleavage-resistant Parkin (118). Accumulated damaged mitochondria produce increased amount of mROS, which allows further inflammasome activation and inflammatory pyroptotic cell death in macrophages and amplification of inflammatory response (118).

In another study, caspase-1 was reported to directly cleave Toll/Interleukin-1 receptor domain-containing adapter-inducing interferon-β (TRIF) and generate TRIF fragments that inhibit the induction of autophagy in macrophages after infection with P. aeruginosa (117). Consistently, expression of cleavage-resistant TRIF increased the autophagy in infected macrophages. Pharmacological inhibition of caspase-1 before *P. aeruginosa* infection in mice lead to increased autophagy in harvested neutrophils and bacterial clearance in peritoneum (117). Caspase-1-mediated TRIF cleavage was confirmed in microglia after NLRP3 inflammasome activation with neurotoxic prion peptide PrP-106-126 and negatively regulates autophagy (114). Furthermore, silencing of NLRP3 or ASC in microglia suppressed caspase-1 activation and enhanced autophagy after PrP-106-126 stimulation (114). Therefore, silencing of NLRP3 inflammasome components in microglia, followed by NLRP3 inflammasome activation leads to enhanced autophagy, most likely due to suppressed caspase-1 activation.

## NLPR3 Inflammasome-Associated Inflammatory Diseases and Autophagy

### Gouty Arthritis

Gouty arthritis is a metabolic disorder caused by excess circulating uric acid that forms uric acid crystals and accumulates in synovial fluids and cartilage, resulting in arthritic inflammation (119). Acute gouty arthritis is characterized by periodic attacks associated by rapid onset pain, swelling, redness of affected joint, fever, and sometimes flu-like symptoms, followed by symptom-free period. The inflammatory response is initiated by formation and deposition of monosodium urate crystals (MSU), causing subsequent release of inflammatory mediators. Over time, periodic acute gout can lead to chronic tophaceous gout, characterized by chronic pain, chronically stiff, and swollen joints, as well as damage to bone and cartilage due to large deposits of crystals (119, 120).

Proinflammatory activity of uric acid crystals depends mainly on the activation of NLRP3 inflammasome and proteolytic activation of IL-1β (121–123). Macrophages from mice deficient in main components of the inflammasome, such as NLRP3, ASC, and caspase-1, have a reduced IL-1β release (122). MSU was shown to increase protein expression of components of NLRP3 inflammasome, including NLRP3 and pro-IL-1β in macrophages and fibroblast-like synoviocytes (124, 125). This suggests that MSU is involved in the priming and activating step of NLRP3 inflammasome activation and may explain why MSU alone can induce the release of IL-1β (125).

The molecular mechanism for MSU-induced NLRP3 inflammasome activation is not yet fully elucidated. Recently, Nomura et al. demonstrated that MSU crystals induced K^+^ efflux, leading to a Ca^2+^ influx-dependent depolarization of mitochondrial membrane potential and decreased intracellular ATP concentration (126). This increased release of IL-1β independently of MSU crystal-induced mitochondrial ROS production (126). Another study by Ives et al. found increased xanthine oxidase (XOR) activity in response to crystalline NLRP3 activators (127). Silencing XOR or pharmacological XOR inhibitors reduced MSU-induced cytoplasmic XOR-derived ROS as well as mitochondrial ROS generation and consequently suppressed the release of IL-1β (127).

As mentioned above, p62 expression negatively regulates NLRP3 activation by mediating mitophagic elimination of damaged mitochondria upon NLRP3 activation (86). Conversely, Jhang et al. proposed that MSU crystal-induced lysosomal disruption and consequent impairment of autophagy leads to accumulation of p62 (128). p62 can interact with Kelch-like ECH-associated protein 1 (Keap-1), which normally acts as a transcriptional repressor by binding to nuclear factor E2-related factor 2 (Nrf2), a transcription factor involved in oxidative stress (129). MSU crystals elevated levels of Keap-1 bound to p62 and released Nrf2 from Keap-1, which facilitated its translocation to the nucleus (128). Nrf2 and its transcriptional products were shown to be required for MSU crystals-induced NLRP3 activation (128). Furthermore, a positive feedback between p62 and ROS generation in MSU crystal-induced inflammation was suggested (130).

AMP-activated protein kinase (AMPK) is a nutritional biosensor, which can alter energy metabolism *via* regulating the expressions or activities of downstream molecules, including a nicotinamide adenine dinucleotide (NAD)-dependent deacetylase sirtuin 1 (SIRT1). SIRT1 was suggested to promote autophagy through regulating autophagy-related genes expressions (131). MSU crystals were shown to decrease AMPKα activity in macrophages by inhibiting its phosphorylation (Thr172) (132, 133). Selective AMPK activator A-769662 increased basal phosphorylation of AMPK, prevented its de-phosphorylation in response to MSU crystals and attenuated MSU crystal-induced IL-1β release in macrophages (132). Similarly, arhalofenate acid also enhanced phosphorylation of AMPKα, expression of SIRT1 and inhibited MSU crystal-induced IL-1β release *in vitro* in macrophages and *in vivo* in murine subcutaneous air pouch model (133). Conversely, AMPKα1 deficiency significantly enhanced inflammatory responses to MSU crystals *in vitro* and *in vivo*, as demonstrated by increased MSU crystal-induced IL-1β release and cleaved caspase-1 AMPKα1-deficient macrophages (132). Furthermore, AMPK activator A-7699662 and arhalofenate acid reduced MSU crystals-induced NF-κB-mediated NLRP3 expression and caspase-1 cleavage, indicating inhibition of NLRP3 inflammasome activation (132, 133). Additionally, arhalofenate prevented MSU crystal-induced mitochondrial damage, such as loss of intact mitochondrial cristae and mitochondrial ROS generation (133).

MSU crystals were demonstrated to induce autophagy, indicated by increased levels of LC3-II and presence of LC3 puncta, which were attenuated by autophagy inhibitor 3-MA in different cell types (113, 133, 134). MSU crystals also induced p62 expression, which was decreased using AMPK activator A-7699662 or arhalofenate acid (133). Therefore, arhalofenate acid promotes autophagy by phosphorylating AMPKα, thereby preventing accumulation of p62 by MSU crystals and inhibiting NLRP3 inflammasome activation (133). Resveratrol is a natural agonist of SIRT1 and was shown to ameliorate the inflammatory response *via* promotion of MSU crystals-induced autophagy in patients with gout. Resveratrol restored SIRT1 protein level that was downregulated following MSU crystal challenge and reduced MSU crystal-induced IL-1β release. Resveratrol induced autophagy, specifically mitophagy, as demonstrated by enhanced levels of LC3-II, increased AMPKα phosphorylation and decreased mTOR phosphorylation (89, 135).

Therefore, MSU crystals induces expression of inflammatory genes (NLRP3, pro-IL-1β), NLRP3 inflammasome activation, and autophagy. As mentioned above, autophagy can regulate NLRP3 inflammasome activation through various mechanisms. Disruption of autophagy through downregulation of proteins with crucial role in autophagy induction, such as AMPKα1, showed increased MSU crystals-induced inflammatory response, whereas induction of autophagy by AMPK activators and SIRT1 agonist demonstrated ameliorated inflammatory response (89, 132, 133, 135). However, MSU crystals can cause lysosomal destabilization and damage, thereby impair of autophagy. Reduced autophagic flux results in accumulation of p62 and excessive p62 stimulates NLRP3 inflammasome activation through Nrf2-mediated transcription and ROS production (128). Since AMPK activators also regulated expression of genes involved in inflammatory pathway, induction of autophagy *via* AMPK activation alleviates the gouty inflammation and appears to be beneficial. Nonetheless, further studies and investigations are required for development of a potential therapeutic strategies for treatment of gouty inflammation.

### Familial Mediterranean Fever

Familial Mediterranean Fever (FMF) is the most common hereditary periodic fever syndrome (HPF). It is caused by mutations in Mediterranean fever (MEFV) gene that encodes protein pyrin (TRIM20) (136, 137). Clinically, it is characterized by recurrent, self-limited attacks of fever, and serositis (138). Pyrin is primarily expressed in monocytes/macrophages and neutrophils. Initially, the disease-causing mutation in pyrin were considered loss-of-function mutations. It was suggested that pyrin interacts with NLRP3 inflammasome component in inhibitory manner and FMF-associated mutations prevent these inhibitory interactions (94, 139). However, further studies have demonstrated that MEFV gene mutations are gain-of-function resulting in the activation of NLRP3 inflammasome (140, 141). Recently, is was suggested that pyrin is a cytosolic PRR and assembles its own inflammasome complex upon recognition of inactivating modification of the RhoA GTPase (142).

RhoA GTPase activates serine/threonine protein kinases PKN1 and PKN2 that directly phosphorylate pyrin (143). Phosphorylated pyrin bind proteins 14-3-3ε and 14-3-3τ, which block pyrin and prevent the formation of active pyrin inflammasome (143). FMF-associated mutations are clustered in carboxy-terminal B30.2 domain of pyrin, which implies that mutations might interfere with regulatory role of this domain (144). Binding of inhibitory protein 14-3-3ε to mutant pyrin was reduced compared to wild-type pyrin (143). It is still unclear how B30.2 domain retains pyrin in its inactive state, however several different hypotheses were suggested and are reviewed by Heilig and Broz (145).

Inflammation in FMF patients is therefore mostly a result of a deregulated activation of pyrin inflammasome. However, other inflammatory pathways and inflammasome complexes might be involved in inflammatory phenotype in FMF patients. FMF-associated mutations disturb self-inhibition of pyrin inflammasome and also reduce removal of NLRP3 by autophagy. As mentioned above, pyrin interacts with NLRP3 inflammasome components and leads to their autophagic interaction, thereby acting as anti-inflammatory factor (95). FMF-associated mutations in pyrin perturb ULK1 recruitment to NLRP3 and diminish autophagic degradation of NLRP3 and hence aggravate inflammatory response in patients with FMF during attacks of fever.

### Sepsis

Sepsis is defined as a life-threatening organ dysfunction caused by a dysregulated host response to infection (146). The host's innate immune system is the first line of defense against infection. At the early stage of sepsis, macrophages secret large amounts of pro-inflammatory cytokines and factors, which exacerbate inflammatory response. Immunosuppression in the late stage of sepsis, due to the excessive apoptosis of macrophages, makes host susceptible to recurrent infections and increases mortality (147). NLRP3 inflammasome activation was implicated in different systems affected by sepsis, including mitochondrial energetic response, cardiovascular system, gastrointestinal system, renal system, respiratory system, and central nervous system (148).

Recently, it has been reported that autophagy influences inflammatory response during sepsis and has a protective role by alleviating organ dysfunction and improving outcomes (149–152). Autophagy plays a protective role in sepsis through various different mechanisms, including clearance of pathogens, dampening of inflammatory response, preventing immunosuppression, and regulating metabolism (153). Autophagy inhibits inflammatory response during sepsis by removing inflammasome activators and degrading inflammasome components and inflammatory cytokines, as described above. Induction of autophagy was shown after a cecal ligation and puncture (CLP)-induced sepsis in mice (150–152). CLP induced an increased LC3-II/LC3-I ratio, an increased number of autophagosomes and their co-localization with lysosomes in different tissues and organs, including liver, lungs, and kidneys, as well as in peritoneal macrophages (150–152).

Induction of autophagy by rapamycin treatment protected mice from renal damage in CLP-induced sepsis and increased survival rate by reducing systemic inflammation (149, 152). Sinomenine hydrochloride (SIN-HCl), which is widely used to treat rheumatoid arthritis, was shown to enhance CLP-induced autophagy in mice (151). SIN-HCl treatment improved the survival of CLP-operated mice and reduced multiple organ dysfunction and attenuated the release of inflammatory cytokines through regulation of autophagy (151). Conversely, inhibition of autophagy by chloroquine, which suppresses fusion of autophagosomes and lysosomes, resulted in greater liver dysfunction and higher mortality rate in CLP-operated mice (150).

Furthermore, LPS-induced sepsis in mice was shown to induce mitophagy, as indicated by greater number of autophagosomes containing only mitochondria in murine hearts following LPS injections compared to controls (154). Sestrins (SESNs) are highly conserved proteins that protect cells exposed to environmental stresses, including oxidative stress, and maintain metabolic homeostasis through regulation of AMPK and mTOR signaling. SESN2 was shown to induce autophagy through activation of AMPK and inhibition of mTOR (155, 156). SESN2 was also demonstrated to be required for mitophagy through mitochondrial priming and autophagosome formation and to suppress prolonged inflammasome activation in macrophages treated with LPS and ATP (87). SESN2-deficient mice had higher serum levels of pro-inflammatory cytokines IL-1β and IL-18, elevated organ dysfunction and higher mortality rate in CLP- and LPS-induced sepsis (87).

Therefore, autophagy has a protective role in sepsis through dampening inflammatory response by removing NLRP3 activator, such as damaged mitochondrial and ROS, as well as degrading NLRP3 inflammasome components. Induction of autophagy results in alleviated organ dysfunction and higher survival rate, whereas inhibition of autophagy accelerates organ dysfunction and reduces survival rate in sepsis. Autophagy is elevated in early stages of sepsis, whereas in late stage of sepsis autophagic activity is suppressed, which is considered a major cause of immune suppression, inflammatory dysregulation, and apoptosis dysfunction (153). This indicates that promoting autophagy might be an effective treatment to relieve organ dysfunction during severe sepsis. However, excessive autophagy during sepsis might lead to imbalance between apoptosis and autophagy, autophagic death of macrophages and consequently immunosuppression (157). Therefore, identifying target molecules for modulating autophagic activity and identifying biomarkers for assessment of autophagy levels *in vivo* is essential for developing treatment. Since balance between autophagy, inflammatory response, and apoptosis is crucial for alleviating organ dysfunction and better outcomes, autophagy inducers could be administrated only at appropriate times, to prevent excessive autophagy and immunosuppression.

## Conclusion

During the past decade, numerous studies have shown interrelation between NLRP3 inflammasome and autophagy. As demonstrated by various studies NLRP3 inflammasome activation can regulate autophagy induction and on the other hand autophagy can control inflammasome activation and suppresses its activity. The two-street regulation provides negative and positive feedback loops necessary for balance between required host defense inflammatory response and prevention of excessive inflammation. Activation of caspase-1 may lead to suppressed autophagy induction to increase inflammatory response, necessary for pathogen removal. However, excessive inflammation can lead to organ and tissue damage and inflammatory diseases and autophagy can dampen the inflammatory response through removal of NLRP3 inflammasome activators and inflammatory components. There seem to be multiple layers of regulation of inflammatory response and interactions between various vital pathways such as autophagy and inflammasome activation can determine cell fate. Induction of autophagy can help cells to survive an inflammatory insult, whereas unrestricted inflammasome activation can result in inflammatory cell death, such as pyroptosis, which can lead to excessive inflammation. The outcome seems to be cell-specific as it can differ in non-inflammatory and inflammatory cells, as well as between different inflammatory cells such as macrophages, dendritic cells, and neutrophils. The specific conditions that induce NLRP3 inflammasome activation and autophagy can also lead to different outcomes, which signifies adaptable response, that we have yet to completely uncover and understand. In the future, manipulating these pathways could be possible treatment for patients suffering from inflammatory, degenerative, or malignant diseases.

## Author Contributions

MB: writing—original draft preparation. NK-J: writing—review and editing, supervision. All authors have read and agreed to the published version of the manuscript.

## Conflict of Interest

The authors declare that the research was conducted in the absence of any commercial or financial relationships that could be construed as a potential conflict of interest.
